# Changes of urinary immunity and microbiome after intravesical BCG therapy and their association with outcomes in NMIBC

**DOI:** 10.37349/etat.2026.1002365

**Published:** 2026-04-13

**Authors:** Yuki Oda, Makito Miyake, Nobutaka Nishimura, Takuto Shimizu, Takuya Owari, Kota Iida, Yasushi Nakai, Nobumichi Tanaka, Kiyohide Fujimoto

**Affiliations:** Queen Mary University of London (QMUL), UK; ^1^Department of Urology, Nara Medical University, Kashihara 634-8521, Japan; ^2^Department of Prostate Brachytherapy, Nara Medical University, Kashihara 634-8521, Japan

**Keywords:** *Bacillus* Calmette–Guérin (BCG) therapy, non-muscle invasive bladder cancer, urinary immune profiling, urinary microbiome dynamics, Firmicutes/Bacteroidetes ratio, progression-free survival

## Abstract

**Aim:**

Intravesical *Bacillus* Calmette–Guérin (BCG) is the standard therapy for non-muscle invasive bladder cancer (NMIBC); however, many patients experience recurrence or progression. We examined how urinary immune signals and the urinary microbiome change across BCG and are related to outcomes.

**Methods:**

In this single-center prospective cohort study, adults with NMIBC underwent transurethral resection of bladder tumor (TURBT), followed by BCG induction. Urine was collected before TURBT, before BCG, after BCG induction, and three months later. Urine sediment mRNA (PD-L1, PD-L2, CD33, and CD204) was quantified using TaqMan ΔCt. The urinary microbiome was profiled using 16S rRNA gene sequencing, and diversity, composition, and taxon balance were evaluated using nonparametric tests, PERMANOVA, repeated-measures correlations, and mixed-effects models. We analyzed the relationship between the urinary microbiome and prognosis.

**Results:**

Twenty-three patients were analyzed; ten recurrences, eight progressions, and three deaths were observed. Relative to baseline, CD33 increased after BCG and after three months; PD-L2 increased immediately after BCG and returned to baseline by three months; PD-L1 and CD204 increased after BCG. Shannon alpha-diversity was unchanged, but total read count was higher at three months, with stable beta-diversity and dispersion. Higher PD-L1 expression was associated with lower Actinobacteria abundance in the bladder cancer microenvironment. A higher post-BCG Firmicutes/Bacteroidetes ratio was associated with worse prognosis, with the clearest signal for progression-free survival (PFS) observed in the univariate Cox models. Higher post-BCG *Corynebacterium* and Enterobacteriaceae abundance was associated with better PFS.

**Conclusions:**

BCG was associated with higher urinary PD-L1/PD-L2 and myeloid marker transcripts, while overall community structure remained stable. These exploratory data support that pre-BCG microbial features may be related to early response, and post-BCG profiles may reflect durability and survival. Urine immune-microbiome profiling could be a feasible, noninvasive adjunct for monitoring and risk stratification in NMIBC.

## Introduction

Bladder cancer is the tenth most common malignancy worldwide, with approximately 570,000 new cases and 210,000 deaths annually [1, 2]. Approximately 75% of cases are non-muscle invasive bladder cancer (NMIBC), for which the therapeutic foundation combines transurethral resection of bladder tumor (TURBT) with intravesical *Bacillus* Calmette–Guérin (BCG) therapy [3–5]. Since its introduction by Morales et al. [3] in 1976, BCG has remained the standard of care for preventing recurrence and progression, yet 30–40% of patients still experience recurrence or disease progression after treatment. This persistent failure rate highlights the urgent need for predictive potential markers and a deeper understanding of host–immune–microbiome interactions.

Most bladder cancers are urothelial carcinomas and are strongly linked to carcinogen exposure, particularly tobacco smoke [6]. Clinically, patients often present with hematuria, and approximately three-quarters of cases are diagnosed as non-muscle-invasive disease [6]. Although NMIBC is associated with favorable cancer-specific survival compared with muscle-invasive disease, it is characterized by frequent recurrence and a prolonged need for risk-adapted surveillance and intravesical therapy, resulting in substantial clinical burden [7]. Current guidelines, therefore, recommend risk stratification based on routinely available clinicopathological features [e.g., stage, grade, concomitant carcinoma in situ (CIS), and prior recurrence] and the use of prognostic models to guide treatment intensity and follow-up [8]. However, prediction at the individual level remains imperfect, and there is an unmet need for noninvasive biomarkers that reflect treatment-induced immune dynamics and identify patients at higher risk of recurrence or progression [6, 8]. These considerations provide a rationale for longitudinal urine-based profiling during BCG therapy.

BCG activates dendritic cells and macrophages via urothelial pathways and induces cytokines such as IL-6, IL-8, and TNF-α that may be associated with innate and adaptive immunity [9–11]. Recently, BCG has been reinterpreted as a model of “trained immunity”, in which innate immune cells undergo epigenetic reprogramming that augments secondary responses [10, 12]. Concurrently, immune regulatory mechanisms, including programmed death-ligand 1 (PD-L1)/PD-L2 upregulation and myeloid-derived suppressor cells (MDSCs) and M2-polarized macrophage induction, are activated and modulate antitumor responses [13–15]. Therefore, understanding the dynamic balance between activation and regulation is central to therapeutic optimization.

The urinary tract was once considered sterile; however, next-generation sequencing identified a resident urinary microbiome in healthy individuals [16–18]. Dysbiosis has been reported in urinary tract infection (UTI), interstitial cystitis, and bladder cancer [19–22], and in NMIBC, taxa such as Enterobacteriaceae, *Corynebacterium*, and *Streptococcus* have been implicated in host immune responses and carcinogenic risk [23]. However, evidence linking broader phylum-level patterns, such as Proteobacteria predominance or the Firmicutes/Bacteroidetes (F/B) ratio, to recurrence or immune homeostasis in the urinary tract remains limited [24–26]. However, most existing studies are cross-sectional, and longitudinal microbiome changes across the BCG course and their temporal relationships with immunity remain insufficiently characterized [23].

Analysis of immune-related gene expression in urine sediment offers a noninvasive window into the bladder’s intravesical immune environment. PD-L1, PD-L2, CD204, and CD33 expression are associated with BCG responsiveness and recurrence [15]. However, no study has evaluated these immune indices and microbiome compositions in a longitudinal and integrated manner. This approach can reveal dynamic immunity-microbiome coupling, informing mechanisms of treatment response and enabling predictive marker development.

Accordingly, we aimed to collect urine from patients with NMIBC at four time points: before TURBT, before BCG induction, immediately after completion of BCG induction, and 3 months thereafter, to concurrently analyze temporal dynamics of urinary immune markers and the urinary microbiome. We hypothesized that intravesical BCG elicits transient yet coordinated changes in the urinary immune environment and microbial community, and that these features are associated with clinical outcomes such as recurrence and progression.

## Materials and methods

### Study design and ethics

A single-center, prospective, observational cohort study (July 2016 to August 2018) was performed. Clinical management adhered to international guidelines. The protocol was approved by the Nara Medical University Ethics Committee (No. 3698). All participants provided written informed consent, and data were anonymized prior to analysis. This study was conducted in accordance with the Declaration of Helsinki.

**Figure 1:**
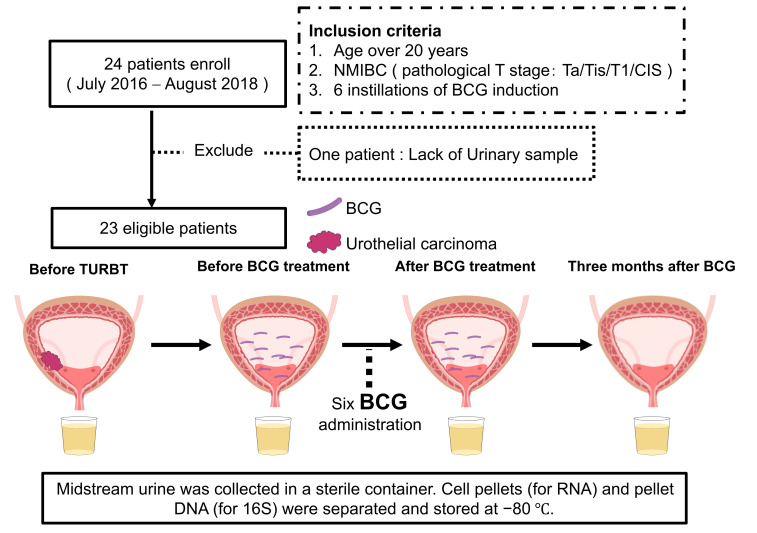
**Schematic design of the study.** Flowchart showing the enrollment of 24 patients with non-muscle invasive bladder cancer (NMIBC), exclusion of one patient because of a lack of urine samples, and inclusion of 23 patients in the final analysis. Midstream urine samples were collected at four time points: before transurethral resection of bladder tumor (TURBT), immediately before *Bacillus* Calmette–Guérin (BCG) induction, immediately after six BCG instillations, and 3 months after completion. Samples were centrifuged and stored at −80°C until analysis.

Adults (> 20 years) with NMIBC (Ta/Tis/T1; CIS permitted) scheduled for TURBT and six instillations of BCG induction were eligible for study inclusion. Patients with ≥ T2 disease, synchronous upper-tract urothelial carcinoma, systemic antibiotic use within 28 days, active UTI, immunodeficiency, or ongoing therapy for another malignancy were excluded. Of the 24 enrolled patients, one was excluded (lack of urine samples), leaving 23 patients for analysis (Figure 1).

### Sampling and immune transcript assays

Urine sediments were collected by centrifugation of midstream urine and stored at −80°C until processing. After thawing on ice, pellets were resuspended in TRIzol LS Reagent (Thermo Fisher Scientific, Waltham, MA, USA) and total RNA was extracted according to the manufacturer’s protocol for liquid samples. Briefly, chloroform was added for phase separation, and the aqueous phase was mixed with ethanol and loaded onto an RNeasy Mini Kit spin column (Qiagen, Hilden, Germany) for purification. Genomic DNA was removed by on-column digestion using the RNase-Free DNase Set (Qiagen). RNA was eluted in RNase-free water, and RNA concentration and purity were assessed spectrophotometrically. Complementary DNA was synthesized from total RNA using SuperScript IV VILO Master Mix (Thermo Fisher Scientific) according to the manufacturer’s instructions. TaqMan assays (Thermo Fisher Scientific, Waltham, MA, USA) quantified PD-L1 (*CD274*; assay ID: Hs00204257_m1), PD-L2 (*PDCD1LG2*; assay ID: Hs00228839_m1), *CD33* (assay ID: Hs01076282_g1), CD204 (*MSR1*; assay ID: Hs00234007_m1), PD-1 (*PDCD1*; assay ID: Hs0001550088_m1), *CTLA4* (assay ID: Hs00175480_m1), *FOXP3* (assay ID: Hs01085834_m1), *CD3E* (assay ID: Hs01062241_m1), *CD14* (assay ID: Hs02621496_s1), CD25 (assay ID: Hs0158122_m1), CD11b (*ITGAM*; assay ID: Hs00167304_m1), and Elastase-1 (assay ID: Hs00204m1) in technical triplicates and were normalized to *GAPDH*. ΔCt was calculated as Ct (target) – Ct (*GAPDH*). Measurements with Ct > 35 or abnormal curves were classified as non-detects, outliers were excluded, and inter-plate correction was applied. We prioritized a urine-sediment mRNA panel because it is feasible with limited sample volume and supports future standardization as a scalable clinical monitoring assay.

### 16S rRNA gene sequencing and bioinformatic analysis

Genomic DNA was extracted from urine sediment pellets and obtained as a purified DNA solution. DNA concentration was measured on a Synergy LX (Agilent Technologies, Agilent BioTek, Santa Clara, CA, USA) using the QuantiFluor dsDNA System (Promega Corporation, Madison, WI, USA). For PCR-based assays, 2 μL of the extracted DNA solution was used as template per reaction in technical quadruplicates, and nuclease-free water was included as a no-template negative control. Amplicon libraries targeting the V3–V4 region of the 16S rRNA gene were prepared via a 2-step tailed PCR workflow, incorporating mixed primers carrying 0–5 random bases to improve initial sequencing base diversity. PCR products were purified after each step using VAHTS DNA Clean Beads (Vazyme Biotech Co., Ltd., Nanjing, China; 1.0× volume). Library concentration was measured on a Synergy H1 (Agilent Technologies) with the same dsDNA assay, and fragment size/quality was assessed using a Fragment Analyzer with the dsDNA 915 Reagent Kit (Agilent Technologies). Libraries were sequenced on an Illumina MiSeq (Illumina, San Diego, CA, USA) using the MiSeq Reagent Kit v3 (Illumina, San Diego, CA, USA; 2 × 300 bp). Reads matching primer sequences were extracted and trimmed (FASTX-Toolkit v0.0.14), low-quality reads (Q  < 20) and pairs < 130 bp were removed with sickle (v1.33) and paired-end reads were merged with FLASH (v1.2.11). Amplicon sequence analysis was performed in QIIME 2 (v2023.7). Demultiplexed paired-end reads were denoised using the DADA2 plugin (qiime dada2 denoise-paired) to infer amplicon sequence variants (ASVs). To remove low-quality leading bases and primer-derived sequence, 25 bp were trimmed from the 5′ end of both forward and reverse reads (trimLeftF = 25; trimLeftR = 25). Reads were truncated at 275 bp (forward) and 205 bp (reverse) (truncLenF = 275; truncLenR = 205), selected based on per-base quality profiles to retain sufficient overlap for paired-end merging. Chimeras were removed using the consensus method (chimera-method = consensus). Unless otherwise specified, remaining DADA2 filtering parameters were left at QIIME 2 defaults. The resulting feature table, representative sequences, and denoising statistics were summarized using qiime feature-table summarize, qiime feature-table tabulate-seqs, and qiime metadata tabulate.

Taxonomy was assigned using the QIIME 2 feature-classifier plugin (classify-sklearn) with a pre-trained Naive Bayes classifier based on the Greengenes 13_8 reference database, trained on the V3–V4 region (341F/805R). Specifically, we used a region-matched Greengenes 13_8 classifier artifact (gg-13-8-99-341-805-nb-classifier.qza). Because Greengenes 13_8 is an older reference database, genus-level assignments may differ under updated databases (e.g., SILVA). Therefore, we limited biological interpretation of taxonomic labels to higher ranks where possible (phylum and family), and we treated genus-level associations as exploratory. Importantly, our primary microbiome inferences were based on compositional log-ratio features (predefined balances and clr/ilr-transformed variables) rather than reliance on a specific taxon label. Representative ASV sequences are provided to enable future reclassification using updated databases. Raw sequencing reads are available in the NCBI Sequence Read Archive (SRA) under BioProject PRJNA1413953.

### Contamination control and filtering for low-biomass urine samples

Urine is a low-biomass specimen and is susceptible to background contamination introduced during sample collection, DNA extraction, PCR amplification, or sequencing. As part of routine quality assurance during library preparation, a no-template negative control (nuclease-free water) was processed in parallel as a PCR negative control. However, sequencing reads for this negative control were not retained/available for the present study. Therefore, we were unable to perform sequencing-based contaminant profiling or apply negative-control-based bioinformatic decontamination workflows, and ASVs were not filtered according to negative-control prevalence. Accordingly, the downstream microbiome analyses should be interpreted as exploratory, acknowledging that low-biomass contamination may have influenced the observed urinary microbiome profiles.

### Statistical analysis

#### Robustness and descriptive summaries for immune transcript data

Because urinary immune transcript measures showed substantial inter-individual variability, we complemented mean ± SD summaries with median [interquartile range (IQR)] at each time point (Table S1). We also visualized patient-level trajectories (spaghetti plots) and overlaid the cohort median trajectory to aid interpretation (Figure S1). To assess the influence of individual participants on key paired comparisons, we performed a leave-one-out (LOO) sensitivity analysis for the before BCG vs. after BCG change: the paired Wilcoxon signed-rank test and the median within-patient change were recalculated after removing one patient at a time, and stability of the direction and statistical significance was examined (Figure S2). These analyses are descriptive robustness checks and do not provide definitive subgroup inference. As a sensitivity analysis addressing cohort heterogeneity, longitudinal trajectories of urinary immune transcripts were visualized as patient-level spaghetti plots stratified by pathologic stage (Ta vs. T1/Tis), tumor grade [low grade (LG) vs. high grade (HG)], and CIS status (CIS vs. non-CIS) (Figure S1).

#### Microbiome statistics

Relative abundances were normalized to the total sequence count. Compositional balances were defined as log-ratios [e.g., Proteobacteria/(Firmicutes + Bacteroidetes), F/B] with pseudo-counts added when necessary. Alpha diversity was quantified using the Shannon index, and beta diversity was assessed using Bray–Curtis distances. Compositional data underwent clr or ilr transformation for balance and regression analyses. Because 16S sequencing yields compositional data, microbiome analyses were performed under a compositional framework. Relative abundances were used for descriptive summaries and visualization. For inferential analyses involving taxa or balances, we used log-ratio approaches: clr transformation was applied to taxon-level abundance vectors, and ilr transformation was applied to predefined balance indices. Total 16S read counts were reported descriptively and were not interpreted as an absolute bacterial load. A complete mapping of each figure/table to the input data scale and transformation is provided in Table S2.

Group differences were analyzed using Kruskal–Wallis and paired Wilcoxon tests with Benjamini–Hochberg correction. Beta-diversity differences were assessed using permutational multivariate analysis of variance (PERMANOVA; ≥ 999 permutations), and dispersion homogeneity was assessed using beta dispersion analysis. Repeated-measures correlation and linear mixed-effects models (immune marker as outcome; microbiome features as main predictors; fixed effects: timepoint, age, sex, antibiotics, UTI, stage, maintenance BCG where available; random intercept: patient) were fitted with Benjamini–Hochberg correction.

#### Prognostic analyses

The TURBT date served as the landmark. The endpoints were recurrence-free survival (RFS), PFS, and overall survival (OS). Kaplan–Meier analyses used predefined quantile-based cutpoint selection for dichotomization (log-rank test). Univariable Cox models treated features as continuous, proportionality was assessed using Schoenfeld residuals, and robust variance was applied when necessary. Analyses were performed in R, and the session details were archived. Given the limited number of events, we performed sensitivity analyses focusing on PFS. We summarized a minimal clinical confounder as a binary risk indicator (high_risk), defined as non-Ta (e.g., T1/Tis) vs. Ta based on pathologic stage. For each post-treatment feature, we fit an adjusted Cox model including high_risk as the only covariate. To mitigate small-sample and separation concerns, we also fit Firth-penalized Cox models. To assess dependence on individual participants, we conducted LOO analyses refitting the adjusted Cox model after excluding one participant at a time and summarizing the resulting hazard ratio range. Finally, we used bootstrap resampling (1,000 iterations) to provide an empirical distribution of hazard ratios and illustrate estimation instability. These analyses were prespecified robustness checks and do not establish definitive prognostic biomarkers.

## Results

### Patient characteristics

Twenty-three patients were included in this analysis. The mean age was 76 ± 8.5 years; 21 of the 23 patients (91%) were men, and 2 (9%) were women. At enrollment, the pathological stage was Ta in 12 patients (52%), T1 in 7 (30%), and Tis in 4 (17%). BCG maintenance therapy was administered to 3 of the 23 patients (13%). After the predefined landmark date, 10 recurrences, 8 progressions, and 3 deaths occurred. Median follow-up was 58.1 months for RFS, 60.5 months for PFS, and 81.8 months for OS (Table 1).

**Table 1:** Patients’ baseline characteristics.

**Variable**	**Overall**
Patients	23
Age (years), mean (SD)	76 (8.5)
Sex (%)	
Female	2 (9)
Male	21 (91)
Height (cm), mean (SD)	165.3 (8.9)
Body weight (kg), mean (SD)	62.5 (12)
Body mass index (kg/m^2^), mean (SD)	23.2 (3.7)
T category (%)	
T1	7 (30)
Ta	12 (52)
Tis	4 (17)
Tumor grade (%)	
HG	11 (48)
LG	12 (52)
Number of tumors (%)	
1	4 (17)
2	13 (57)
3	2 (9)
4	4 (17)
Longest diameter (cm), mean (SD)	3.0 (1.4)
CIS (%)	5 (22)
Maintenance of BCG (%)	3 (13)
Overall follow-up period (month), median (IQR)	89 [57, 108.0]
Overall survival (%)	3 (13)
Overall survival (month), median (IQR)	81.8 [52.2, 99.1]
Recurrence (%)	10 (44)
Recurrence-free survival (month), median (IQR)	58.1 [17.9, 85.1]
Progression (%)	8 (35)
Progression-free survival (months), median (IQR)	60.5 [46.4, 86.6]

Demographic and clinicopathological characteristics of the 23 patients with non-muscle invasive bladder cancer (NMIBC) included in the analysis. Data are presented as mean ± standard deviation (SD) for continuous variables and number (percentage) for categorical variables unless otherwise indicated. Survival times are reported as median [interquartile range (IQR)]. BCG: *Bacillus* Calmette–Guérin; HG: high grade; LG: low grade.

### Longitudinal changes in urinary immune markers

**Figure 2:**
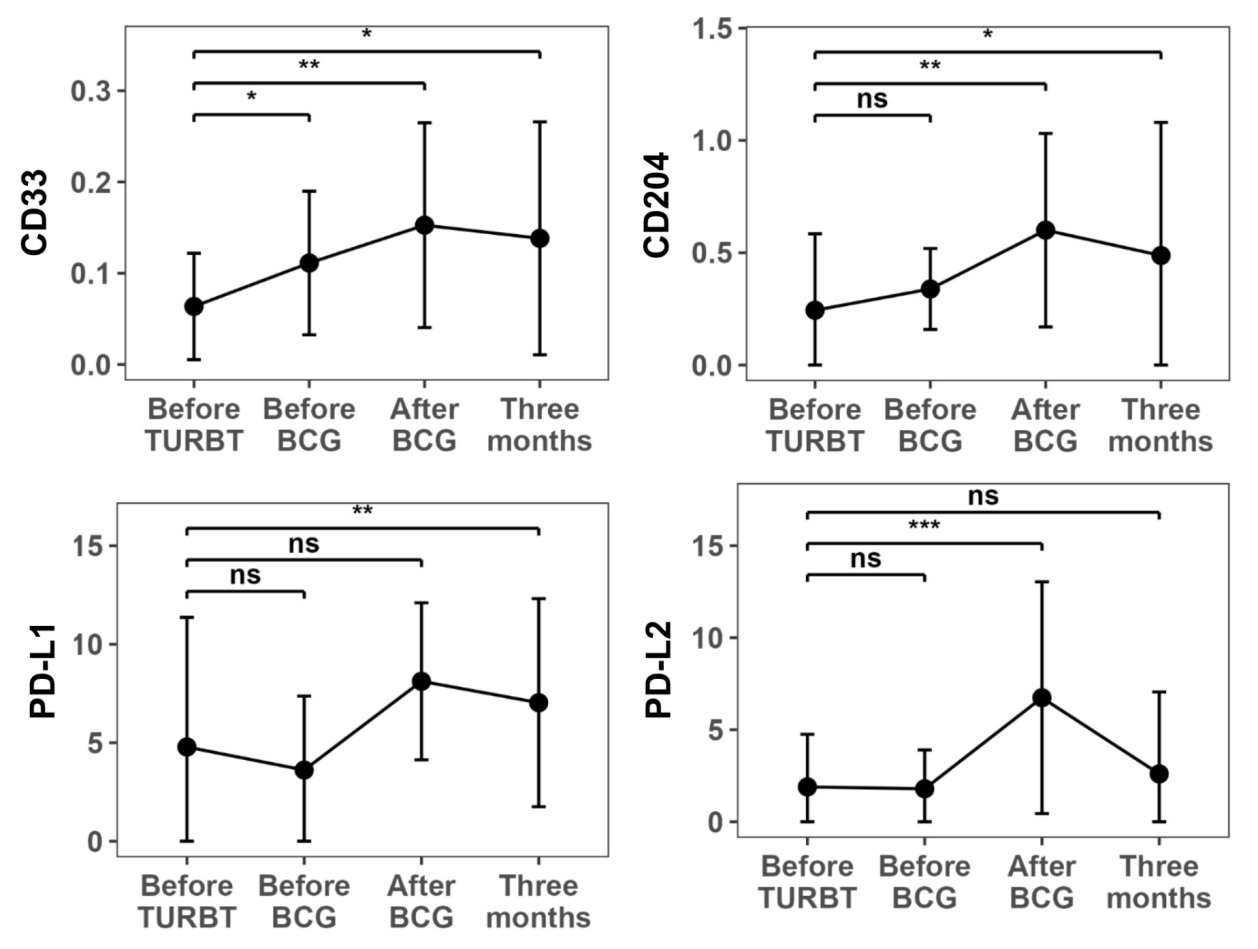
**Time-course changes in mRNA expression of immune cell markers in urine.** ΔCt values (relative to *GAPDH*) for *CD33*, PD-L1 (*CD274*), PD-L2 (*PDCD1LG2*), and CD204 (*MSR1*) in urine sediment collected at four time points: before TURBT, before BCG induction, immediately after completion of six BCG instillations, and 3 months after BCG. *CD33* increased immediately after BCG and remained elevated for 3 months, whereas CD204 significantly increased after BCG. PD-L1 and PD-L2 showed significant increases immediately after BCG treatment, with PD-L2 returning to baseline by 3 months. *P* values were obtained from paired Wilcoxon signed-rank tests versus the pre-TURBT baseline. ns: not significant; *P < 0.05; **P < 0.01; ***P < 0.001. TURBT: transurethral resection of bladder tumor; BCG: *Bacillus* Calmette–Guérin.

Time-point-specific ΔCt values for each immune-related gene are summarized in Table 2. Using the pre-TURBT time point as the reference, paired tests revealed significant post-treatment changes in PD-L1, PD-L2, *CD33*, and CD204 expression. *CD33* levels increased immediately after BCG and remained elevated at 3 months. PD-L2 levels increased significantly immediately after BCG and returned to baseline by 3 months. PD-L1 showed significant differences at one or more post-BCG time points, and CD204 significantly increased after BCG administration (Figure 2). In contrast, PD-1, *FOXP3*, *CD3E*, CD11b, *CD14*, CD25, and Elastase-1 levels showed no significant temporal change (Figure S3, Table S3).

**Table 2:** mRNA expression levels of immune cells in urine sediments at each of four time points.

**Target**	**RNA expression in urine sediments (TaqMan assay ID)**		**Pre-TURBT**	**Before BCG**	**After BCG**	**3 months**
MDSC	*CD33*	Mean ± SD	0.064 ± 0.058	0.11 ± 0.079	0.15 ± 0.11	0.14 ± 0.13
		*P* value (vs. baseline)	Baseline	0.047	0.039	0.047
Immune checkpoint	PD-L1 (*CD274*)	Mean ± SD	4.8 ± 6.6	3.6 ± 3.8	8.1 ± 4.0	7.0 ± 5.3
		*P* value (vs. baseline)	Baseline	0.7	0.065	0.01
	PD-L2 (*PDCD1LG2*)	Mean ± SD	1.9 ± 2.9	1.8 ± 2.1	6.7 ± 6.3	2.6 ± 4.5
		*P* value (vs. baseline)	Baseline	0.94	0.0015	0.45
Macrophage	CD204 (*MSR1*)	Mean ± SD	0.24 ± 0.34	0.34 ± 0.18	0.60 ± 0.43	0.49 ± 0.59
		*P* value (vs. baseline)	Baseline	0.088	0.007	0.032

Mean ± SD ΔCt values (relative to *GAPDH*) for *CD33*, PD-L1 (*CD274*), PD-L2 (*PDCD1LG2*), and CD204 (*MSR1*) in urine sediment collected before TURBT, before BCG induction, immediately after completion of six BCG instillations, and 3 months after BCG. *P* values are from paired Wilcoxon signed-rank tests comparing each time point with the pre-TURBT baseline. Lower ΔCt values indicate higher transcript abundance. MDSC: myeloid-derived suppressor cell; TURBT: transurethral resection of bladder tumor; BCG: *Bacillus* Calmette–Guérin.

Given the heterogeneity of clinicopathologic features in this cohort, we performed a stratified sensitivity visualization. Patient-level trajectories showed that the overall direction of the main longitudinal changes was broadly similar across Ta vs. T1/Tis, LG vs. HG, and CIS vs. non-CIS strata, although subgroup sizes were small and these analyses are descriptive (Figure S1). Given the large dispersion of expression values, we additionally summarized immune transcript levels using median (IQR) at each time point (Table S1), which showed patterns consistent with the main paired-test results based on ΔCt values (Table 2). To evaluate whether statistically significant findings were driven by a small number of influential individuals, we conducted an LOO sensitivity analysis for the before BCG vs. after BCG comparison. The direction of change for PD-L1, PD-L2, and CD204 remained consistent across iterations, and significance for these markers was not dependent on any single patient. In contrast, changes in markers with smaller effect sizes should be interpreted cautiously, given the variability.

### Analysis of microbiota diversity

**Figure 3:**
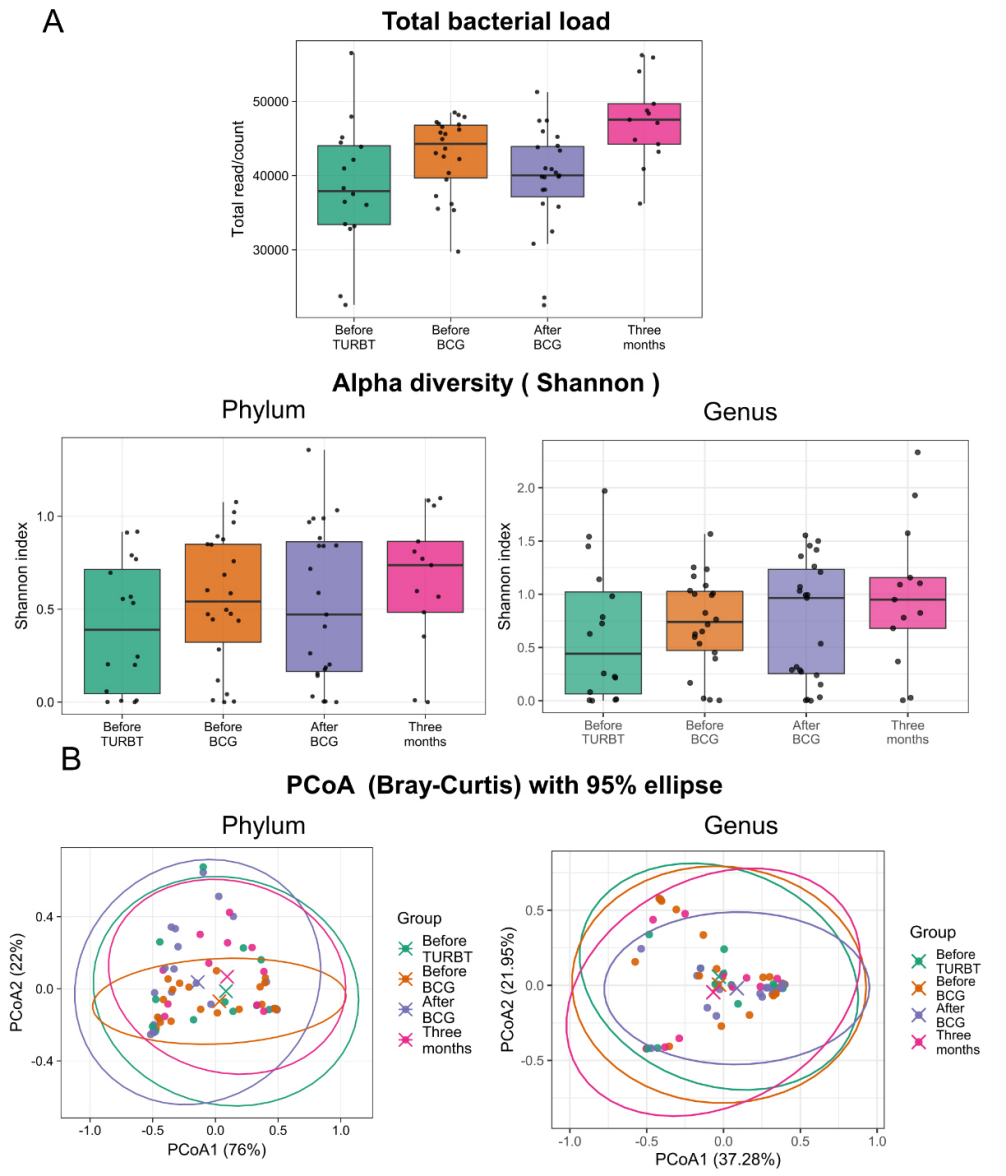
**Urinary microbiome analysis: alpha-diversity and beta-diversity.** (**A**) Alpha diversity quantified by the Shannon index and total 16S rRNA read counts at four time points. Shannon diversity did not significantly differ between time points (Kruskal–Wallis *P* = 0.39), whereas total read counts were higher 3 months after BCG than before TURBT (*q* = 0.011). Read counts do not directly quantify absolute bacterial load and can be influenced by technical factors. Shannon diversity was calculated directly from the filtered count table without rarefaction. (**B**) Beta diversity (Bray–Curtis) of the urinary microbiome by principal coordinates analysis (PCoA) across four time points. PERMANOVA pseudo-*F* = 1.71, *R*^2^ = 0.068, *P* = 0.245. Within-group beta dispersion did not differ between groups (beta dispersion *P* = 0.641). Each point represents a urine sample. Bray–Curtis dissimilarities were computed on relative abundances (total-sum scaling) and tested by PERMANOVA using permutations constrained by participant ID. TURBT: transurethral resection of bladder tumor; BCG: *Bacillus* Calmette–Guérin.

Alpha diversity assessed by the Shannon index did not differ among time points (Kruskal–Wallis, *P* = 0.39). Total 16S read counts can be influenced by sequencing depth, library preparation, and amplification efficiency; therefore, read count differences should not be interpreted as changes in absolute bacterial load without independent absolute-quantification methods. Total 16S read counts differed across time points (*P* = 0.0023) and were higher at three months after BCG than before TURBT (*q* = 0.011; Figure 3A). Beta diversity based on Bray–Curtis distances suggested no clear separation between groups (PERMANOVA pseudo-*F* = 1.71, *R*^2^ = 0.068, *P* = 0.245), and within-group dispersion did not differ (*P* = 0.641; Figure 3B). These findings suggest that the qualitative composition of the urinary community was broadly stable across the four time points. Because total read counts can be influenced by sequencing depth and amplification efficiency, we interpret read count differences as technical or biological variation in sequencing output rather than evidence of changes in absolute bacterial load.

### Microbiota composition (phylum and genus) and balance indices

Taxon labels are shown as assigned by Greengenes 13_8 and should be interpreted as exploratory, particularly at the genus level.

Dominant phyla included Proteobacteria, Firmicutes, Bacteroidetes, and Actinobacteria. Only Bacteroidetes suggested a significant overall effect on relative abundance (*χ*^2^ = 11.0, degrees of freedom = 3, *P* = 0.013) when temporal changes were evaluated using the Friedman test. However, Benjamini–Hochberg-adjusted pairwise comparisons were not significant (minimum *q* = 0.29, for after BCG vs. 3 months and before TURBT vs. 3 months). The remaining phyla (Actinobacteria, Firmicutes, and Proteobacteria) suggested no significant time-dependent differences (all *P* > 0.1).

**Figure 4:**
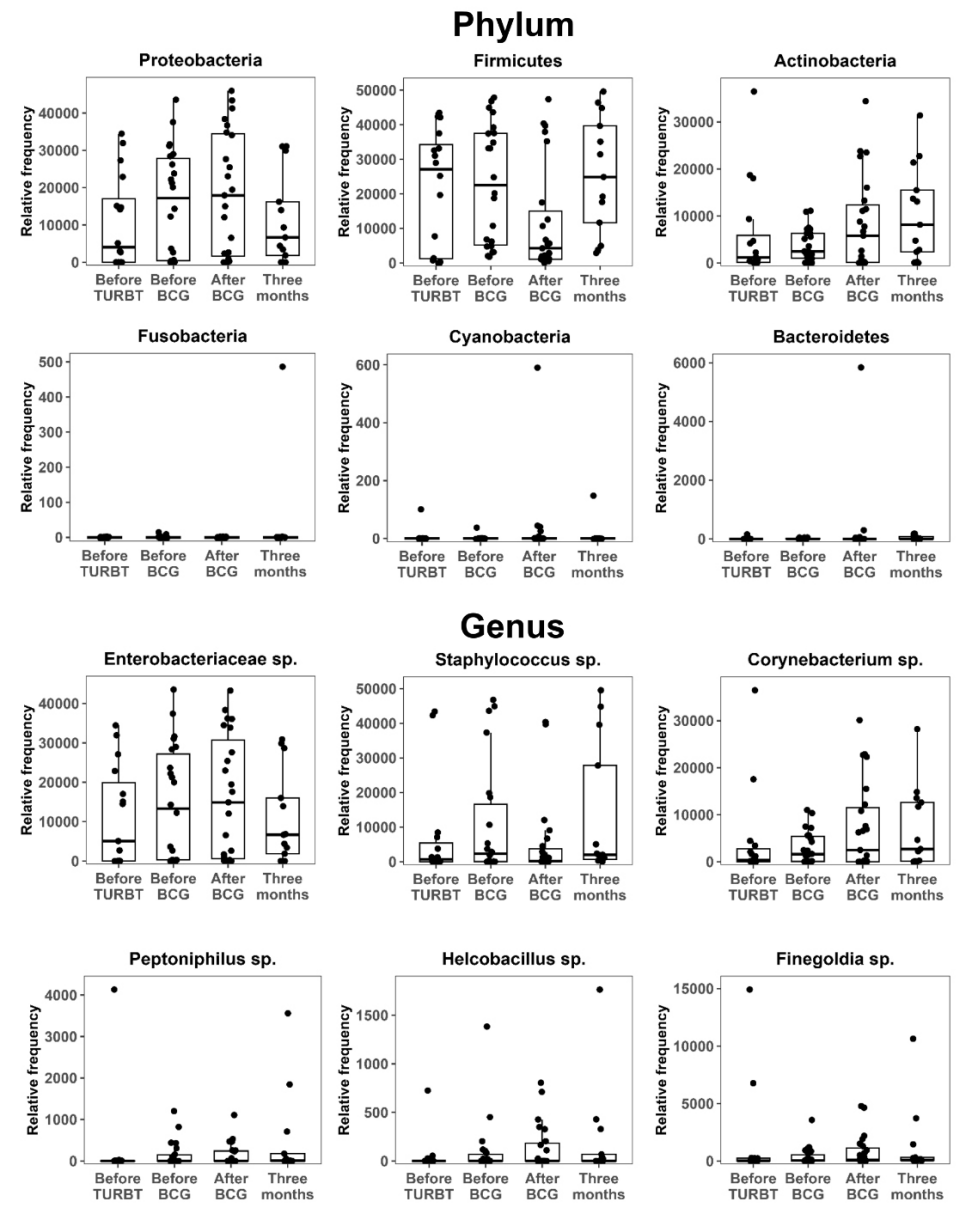
**Urinary microbiome dynamics: phylum and genus.** Relative abundances of dominant bacterial phyla and selected genera in urine samples collected before TURBT, before BCG induction, immediately after BCG completion, and 3 months after BCG. Proteobacteria, Firmicutes, Bacteroidetes, and Actinobacteria dominated throughout; Actinobacteria increased over time, Firmicutes decreased immediately after BCG and recovered by 3 months, and Proteobacteria peaked around BCG initiation. Friedman tests suggested a modest overall temporal effect for Bacteroidetes, although no pairwise comparison remained significant after Benjamini–Hochberg correction. Phylum-level balance indices, such as the F/B ratio, remained stable. TURBT: transurethral resection of bladder tumor; BCG: *Bacillus* Calmette–Guérin; F/B: Firmicutes/Bacteroidetes.

As illustrated in Figure 4, Actinobacteria gradually increased from before TURBT to 3 months after BCG, Firmicutes declined immediately after BCG and recovered by 3 months, and Proteobacteria peaked during BCG initiation. Phylum-level balance indices—F/B ratio, Proteobacteria/Firmicutes + Bacteroidetes, or Proteobacteria/Actinobacteria—did not differ significantly across time points (*P* = 0.68, 0.92, and 0.52, respectively). Collectively, apart from short-term fluctuations in Bacteroidetes, the overall urinary microbiome composition remained relatively stable throughout BCG therapy. The stability of the F/B ratio and Proteobacteria-related balances suggested a maintained equilibrium between inflammation-associated and potentially immunoregulatory microbial consortia.

### Immune-microbiota associations

**Figure 5:**
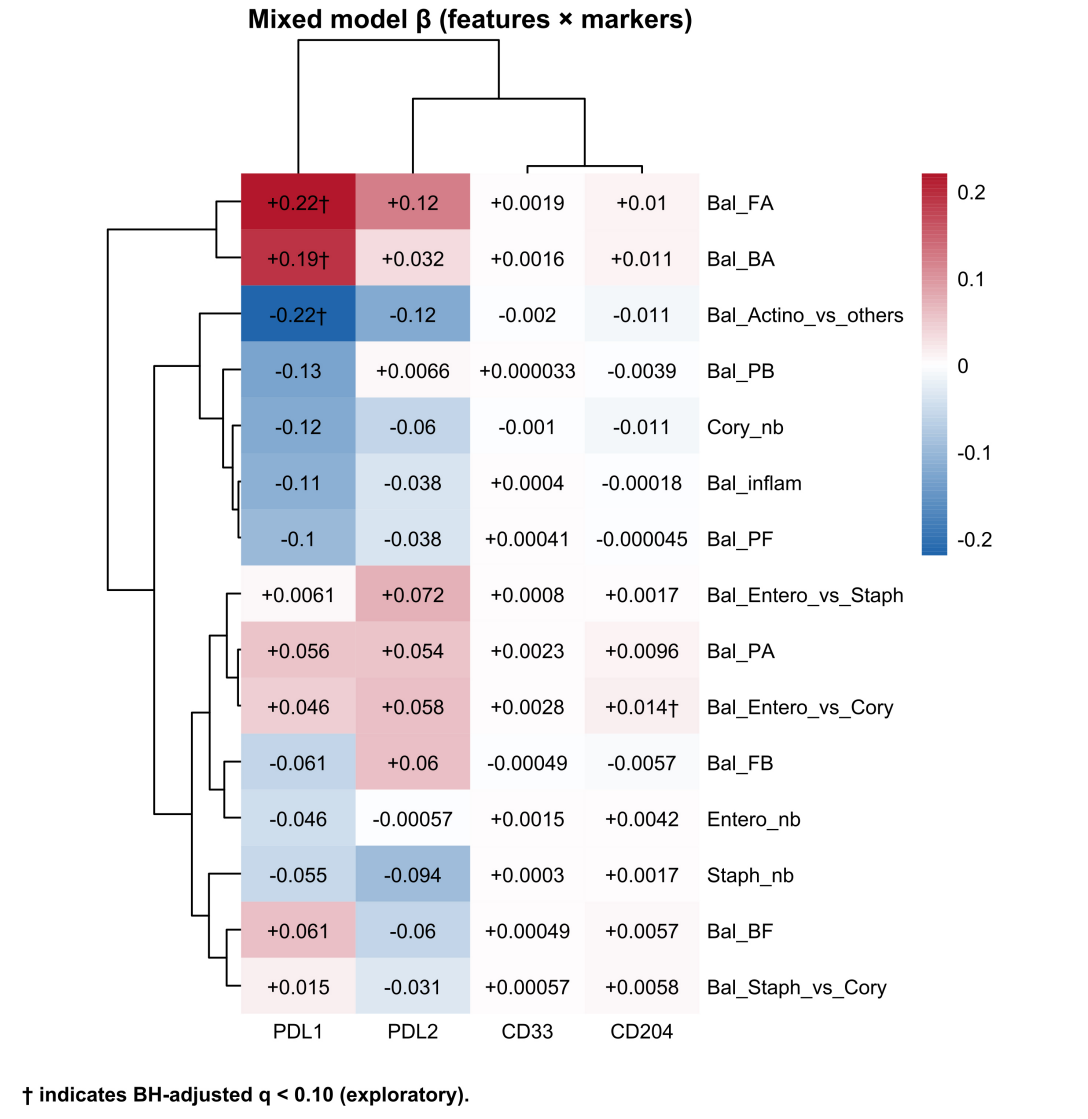
**Urinary microbiome-immune coupling.** Matrix plot summarizing associations between immune-related gene expression *(PD-L1*, *PD-L2*, *CD33*, and *CD204*) and microbiome indices, including phylum-level balances (Bal_) and taxon-specific log-abundances (_nb), across all four time points. Colors indicate the direction and magnitude of the mixed-effects model coefficient (*β*), and numbers in each cell show *β* (two significant digits). Coefficients (*β*) are from linear mixed-effects models with immune transcript levels (ΔCt) as outcomes and microbiome features as predictors, including a random intercept for participant. Taxon-specific features (nb) were entered as clr-transformed abundances (pseudocount = 1), whereas balance indices (Bal) were entered as predefined log-ratio/ilr features; fixed effects included timepoint and available clinical covariates (age, sex, antibiotics, UTI, stage, maintenance BCG). The symbol † indicates associations that met the exploratory false discovery rate threshold (Benjamini–Hochberg adjusted *q* < 0.10). Bal_ denotes the log-ratio between two taxonomic groups, and _nb denotes the log-abundance of a specific taxon. Bal_inflam = log[P/(F + B)]. P: Proteobacteria; F: Firmicutes; B: Bacteroidetes; A: Actinobacteria; Cory: *Corynebacterium*; Staph: *Staphylococcus*; Entero: Enterobacteriaceae; BCG: *Bacillus* Calmette–Guérin; UTI: urinary tract infection. The underlying numerical results (*β*, *P*, and *q* values) are provided in Table S4.

In mixed-effects models, PD-L1 expression was independently associated with several Actinobacteria-related balance indices and remained below an exploratory false discovery rate threshold of 0.10 (*q* = 0.071) (Figure 5). Specifically, PD-L1 was negatively associated with Actinobacteria vs. other phyla (*β* = −0.22, 95% CI −0.38, −0.07, *P* = 0.0059, *q* = 0.071) and positively associated with the Firmicutes/Actinobacteria (*β* = +0.22, 95% CI 0.07–0.36, *P* = 0.0048, *q* = 0.071) and Bacteroidetes/Actinobacteria balance (*β* = +0.19, 95% CI 0.07–0.31, *P* = 0.0023, *q* = 0.0708). CD204 expression was positively associated with the Enterobacteriaceae/*Corynebacterium* balance (*β* = +0.014, 95% CI 0.0049–0.024, *P* = 0.0037, *q* = 0.071). These relationships suggested that Actinobacteria decrease or Firmicutes and Bacteroidetes predominance over Actinobacteria may be linked to PD-L1 induction, whereas increased gram-negative-derived signals may be associated with higher CD204 expression, reflecting an M2-like/phagocytic macrophage axis.

Table S4 contains the full mixed-effects model results (*β*, nominal *P* values, and Benjamini–Hochberg adjusted *q* values) for all immune marker-microbiome feature pairs used to generate Figure 5.

### Prognostic analyses

#### Preoperative (before BCG)

In the log-rank test for RFS, the pre-BCG *Corynebacterium* index (Pre_Cory_nb) significantly stratified recurrence risk (*P* = 0.0026). However, in univariate Cox analyses, no preoperative microbiome indices were significantly associated with OS, RFS, or PFS. These findings suggested that higher preoperative *Corynebacterium* abundance may influence the initial antitumor immune response after BCG treatment (Figure S4), although this effect was not independently captured as a continuous predictor in this small cohort.

#### Post-BCG (after BCG)

**Figure 6:**
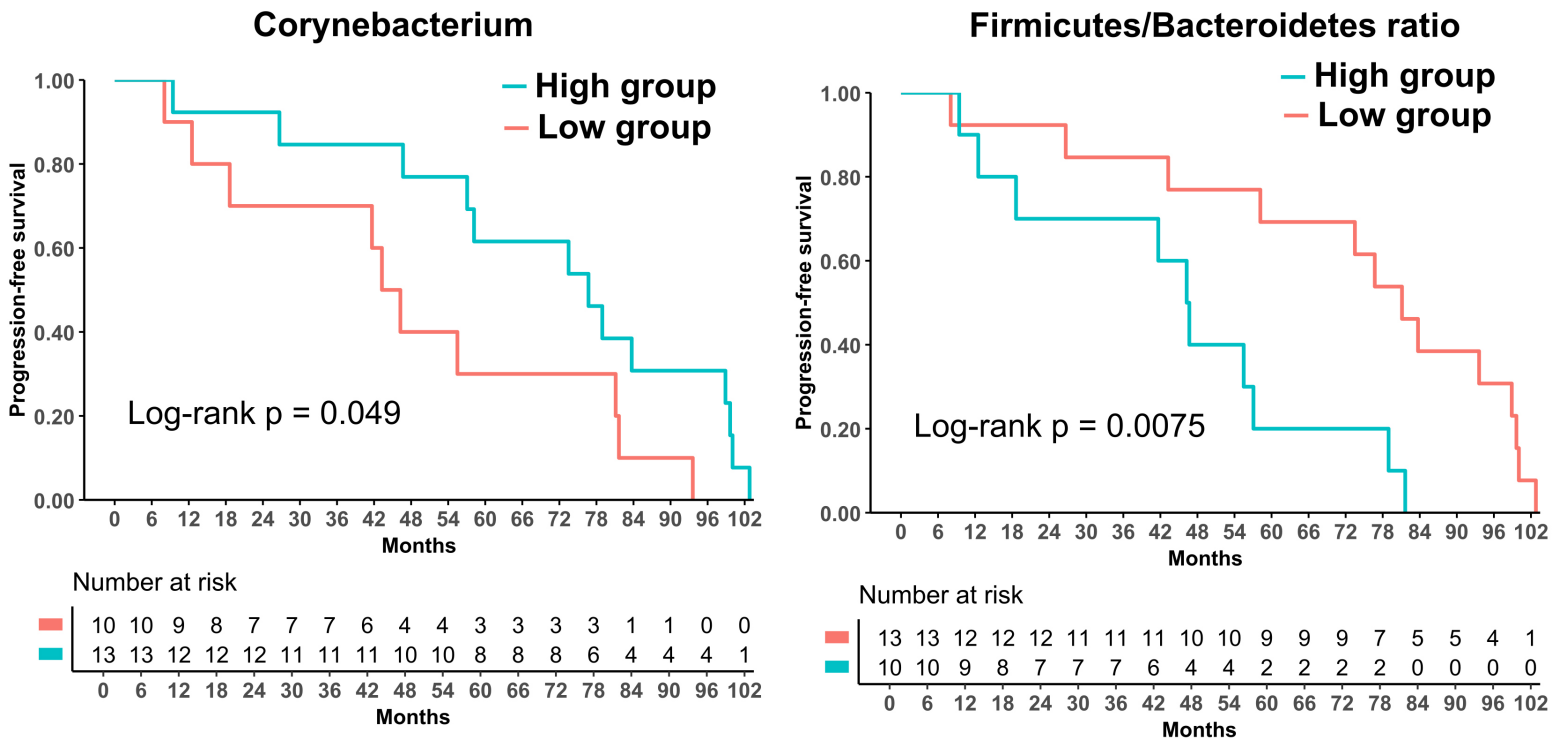
**Prognosis analysis: after and three months after BCG treatments.** Progression-free survival (PFS) curves stratified by quantile-based high versus low groups for post-BCG F/B ratio (Post_Bal_FB), and *Corynebacterium* log-abundance (Post_Cory_nb). Patients with a higher post-BCG F/B ratio experienced poorer PFS, whereas those with higher post-BCG *Corynebacterium* abundance suggested more favorable PFS. *P* values were obtained from log-rank tests. BCG: *Bacillus* Calmette–Guérin; F/B: Firmicutes/Bacteroidetes.

After surgery and BCG exposure, the post-BCG F/B ratio (Post_Bal_FB) was associated with unfavorable outcomes across all three endpoints (RFS, PFS, and OS) in the log-rank analyses (Figure 6). However, in univariate Cox regression models treating the same indices as continuous variables, Post_Bal_FB remained significantly associated only with PFS (HR = 1.55, 95% CI 1.01–2.30; *P* = 0.044). Higher post-BCG *Corynebacterium* (Post_Cory_nb) and Enterobacteriaceae abundances (Post_Entero_nb) were associated with a lower progression risk (HR = 0.61, 95% CI 0.39–0.97; *P* = 0.036; and HR = 0.54, 95% CI 0.33–0.89; *P* = 0.016, respectively; see Table 3).

**Table 3:** Univariate analysis of urinary microbiome parameters before and after BCG treatment and their association with OS, RFS, and PFS.

**Parameters**	**Treatment of BCG**	**Group ratio**	**Univariate**								
			**OS**			**RFS**			**PFS**		
			**HR**	**95% CI**	***P*-value**	**HR**	**95% CI**	***P*-value**	**HR**	**95% CI**	***P*-value**
Bal_FB	Before	High/Low	1.23	0.85–2.04	0.34	1.32	0.85–2.04	0.22	1.28	0.81–2.03	0.3
Cory_nb	Before	High/Low	0.84	0.54–1.29	0.42	0.76	0.50–1.13	0.18	0.76	0.47–1.24	0.27
Entero_nb	Before	High/Low	1.12	0.73–1.72	0.59	0.91	0.60–1.38	0.66	1.37	0.81–2.30	0.24
Bal_inflam	Before	High/Low	1.08	0.74–1.58	0.69	0.97	0.69–1.37	0.87	1.2	0.80–1.79	0.38
Staph_nb	Before	High/Low	0.93	0.62–1.40	0.72	0.81	0.53–1.25	0.34	0.77	0.51–1.17	0.23
Bal_FB	After	High/Low	1.57	0.98–2.50	0.058	1.26	0.82–1.93	0.29	1.55	1.01–2.3	0.044
Cory_nb	After	High/Low	1.12	0.72–1.75	0.61	0.96	0.61–1.51	0.86	0.61	0.39–0.97	0.036
Entero_nb	After	High/Low	0.84	0.54–1.30	0.44	0.66	0.41–1.06	0.087	0.54	0.33–0.89	0.016
Bal_inflam	After	High/Low	1.24	0.69–2.20	0.47	1.3	0.73–2.31	0.37	1	0.60–1.66	0.99
Staph_nb	After	High/Low	1.19	0.77–1.83	0.44	1.06	0.69–1.64	0.78	1.25	0.81–1.93	0.32

Hazard ratios (HRs) and 95% confidence intervals (CIs) from univariable Cox proportional hazards models evaluating the associations of urinary microbiome indices before and after BCG with overall survival (OS), recurrence-free survival (RFS), and progression-free survival (PFS). Each index was modeled as a continuous variable, and HRs represent the change in event hazard associated with a 1-unit increase in the transformed value. Bal_ denotes log-ratio balances between two taxonomic groups, and _nb denotes the log-abundance of a specific taxon. Bal_inflam = log[P/(F + B)]. P: Proteobacteria; F: Firmicutes; B: Bacteroidetes; Cory: *Corynebacterium*; Staph: *Staphylococcus*; Entero: Enterobacteriaceae.

Sensitivity analyses (adjusted Cox with high_risk, Firth penalization, LOO influence, and bootstrap intervals) are provided in Table S5 and Figure S2.

#### Sensitivity analyses for small-sample bias and confounding

Because the number of PFS events was limited and missing post-treatment samples reduced the analyzable set, we performed prespecified sensitivity analyses for the post-treatment features at three months (*n* = 13; events = 4). We fit (i) an adjusted Cox model including a minimal clinical confounder summarized as a binary risk indicator (high_risk: non-Ta vs. Ta), (ii) a Firth-penalized Cox model, (iii) LOO influence analyses, and (iv) bootstrap resampling (1,000 iterations) to illustrate estimate instability (Figure S5).

In the adjusted Cox model, higher Post_Bal_FB showed a consistent direction toward higher progression risk (HR = 1.8, 95% CI 0.90–3.5; *P* = 0.10), and LOO estimates remained > 1 (HR range 1.5–3.7) (Figure S5). Post_clr_*Corynebacterium* showed a consistent protective direction in the adjusted Cox model (HR = 0.59, 95% CI 0.31–1.1; *P* = 0.12), and LOO estimates remained < 1 (HR range 0.26–0.73). In contrast, Post_clr_Enterobacteriaceae was not stable: LOO analyses showed sign flipping with HRs spanning 0.87–1.4. Bootstrap intervals were extremely wide for all three features, highlighting substantial uncertainty and reinforcing that these survival associations should be interpreted as hypothesis-generating rather than confirmatory.

## Discussion

In this prospective pilot study, we used serial urine samples to characterize how intravesical BCG shapes both the urinary immune transcriptome and urinary microbiome in patients with NMIBC. The primary objective was to describe longitudinal changes in urinary immune markers and microbiome features during the BCG course, whereas associations between immune markers and microbiome balance and exploratory prognostic analyses for RFS, PFS, and OS were secondary and hypothesis-generating.

Given the single-center design and limited sample size, our analyses should be interpreted as exploratory and hypothesis-generating rather than definitive evidence of clinical utility or causality. Accordingly, observed microbiome-outcome associations may reflect underlying host inflammatory states and require independent validation. We selected urine sediment mRNA profiling as an immune readout because urine protein measurements can be influenced by dilution and degradation, whereas cellular transcripts from sediments provide a practical multi-target readout of immune activity in a longitudinal sampling design. Nonetheless, protein-level validation will be important in future studies.

Our immune marker data broadly align with current hypothesis-generating models of BCG activity in the bladder. Intravesical BCG instillation leads to bacillus uptake via urothelial, macrophage, and dendritic cells, triggering cytokine and chemokine cascades that recruit and activate innate and adaptive effector cells in the bladder wall [27–29]. This includes robust myeloid activation, TNF-α and IFN-γ production, and subsequent T- and natural killer cell-mediated antitumor activity [27, 28]. Within this context, PD-L1 and PD-L2 upregulation represent an adaptive feedback mechanism that restrains ongoing inflammation; however, it may also dampen antitumor immunity. Increased PD-L1 expression in high-risk NMIBC after BCG has been linked to PD-L1-mediated adaptive resistance as a mechanism of BCG failure [30, 31]. Our finding that urinary PD-L1 and PD-L2 transcripts increased after BCG, alongside enrichment of CD33 and CD204 transcripts as indicators of myeloid activation, supported the concept that BCG creates a dynamic, checkpoint-regulated inflammatory niche at the urothelial surface rather than a static on/off state. Although subgroup sizes were underpowered for definitive inference, stratified spaghetti plots suggested that the main within-patient longitudinal patterns were not driven by a single clinicopathologic subgroup, supporting cautious interpretation as hypothesis-generating (Figure S1).

At the microbiome level, we observed no major shifts in alpha or beta diversity across the four time points; however, total 16S read counts were higher at three months than at baseline. This pattern suggested that the overall configuration of the urinary community is relatively stable; however, BCG was associated with higher total 16S read counts at three months. Read counts do not quantify absolute bacterial load. Previous studies on the bladder microbiome in NMIBC have reported inconsistent findings, with some studies noting modest changes in richness or specific taxa during BCG therapy and others detecting minimal global restructuring compared with non-oncological controls [19, 21, 32, 33]. Moreover, exploratory clinical data have suggested that baseline or on-treatment urinary microbiome features may influence BCG responsiveness [21]. Against this heterogeneous background, our longitudinal within-patient data may support the notion that BCG superimposes inflammatory pressure on a conserved urinary microbiome rather than fully remodeling the community in most patients.

Mixed-effects analyses provide an initial view of how this community structure may both influence and be shaped by the immune response. PD-L1 expression was inversely associated with the overall Actinobacteria abundance and positively associated with the Firmicutes- or Bacteroidetes-dominant balance over Actinobacteria. This pattern parallels immuno-oncology findings in which compositional shifts among major phyla, including the F/B ratio, have been linked to the efficacy of immune checkpoint blockade and systemic inflammatory tone in other cancers [34–37]. Although causality cannot be inferred in this small cohort, one interpretation is that Actinobacteria-rich communities exert more restrained or tolerogenic signaling, whereas Firmicutes- and Bacteroidetes-dominated communities favor a more inflamed, PD-L1-inducing environment at the urothelial surface. Concurrently, CD204 expression, marking phagocytic and M2-like macrophages, was monitored with an Enterobacteriaceae/*Corynebacterium* balance index, suggesting gram-negative-derived signals may influence the CD204-positive myeloid compartment in the BCG-treated bladder.

Our exploratory survival analyses suggested a second layer of complexity: acute immune-microbiome interactions are “frozen in” as more stable post-BCG steady states that may be associated with long-term oncologic outcomes. At baseline, higher preoperative *Corynebacterium* abundance stratified RFS in Kaplan–Meier analysis, although this association did not yield a significant continuous predictor in univariate Cox models, likely reflecting limited power. However, after BCG, the post-BCG F/B ratio consistently correlated with adverse RFS, PFS, and OS, and higher post-BCG *Corynebacterium* and Enterobacteriaceae abundance was associated with better PFS. Accordingly, the association between a higher post-BCG F/B ratio and poor prognosis was most evident for PFS in the Cox regression analyses. These findings parallel gut microbiome studies in which specific consortia and phylum-level ratios, including the F/B ratio, have been associated with immune checkpoint inhibitor response and host inflammatory tone [34–37]. In our cohort, a lower post-BCG F/B ratio together with persistent *Corynebacterium* and Enterobacteriaceae signals may indicate a reconstituted yet balanced urinary ecosystem that supports durable disease control, whereas a persistently high F/B ratio may reflect a dysbiosis state that showed patterns consistent with BCG-induced immunity.

**Figure 7:**
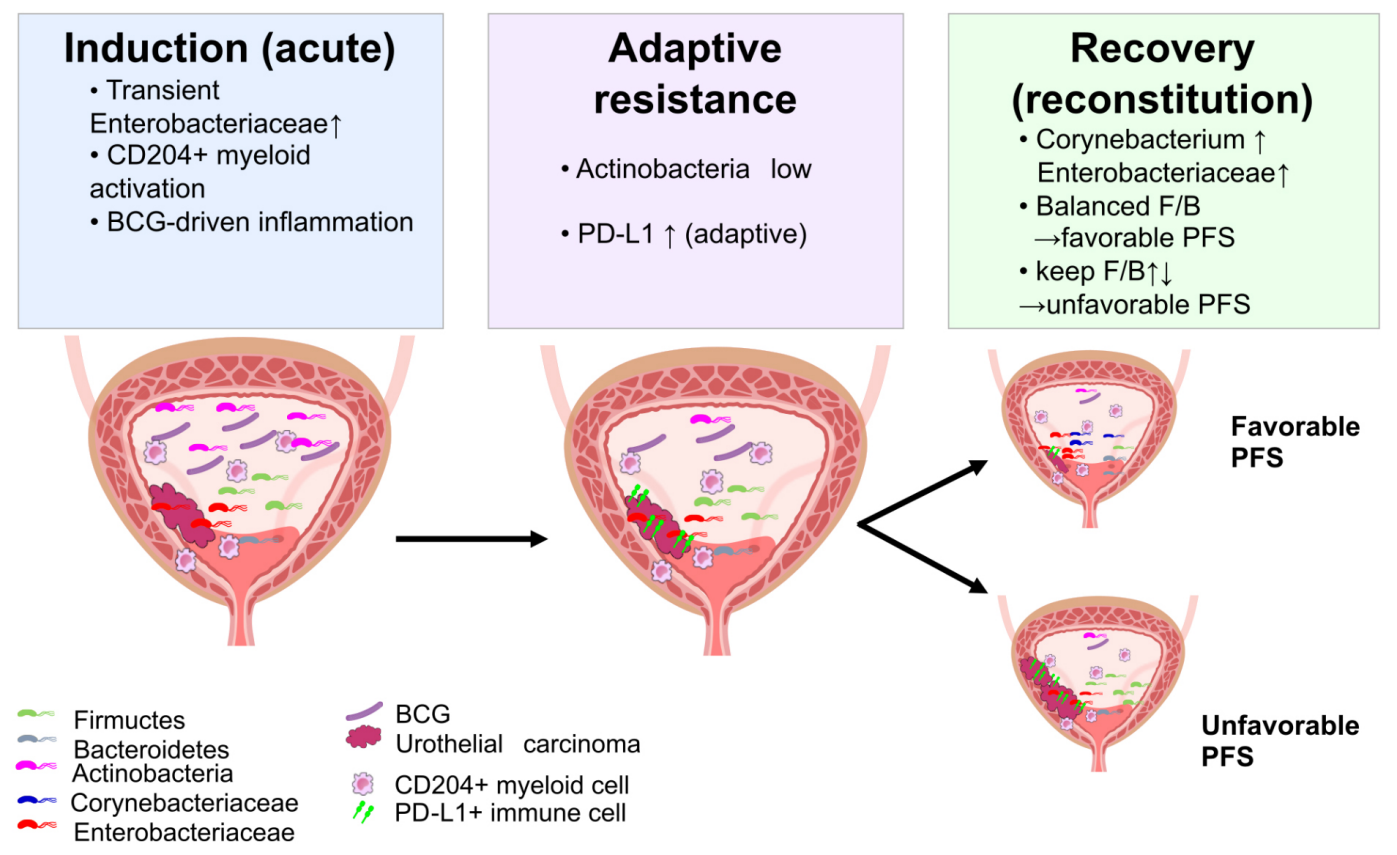
**Proposed working model linking BCG-associated inflammation, urinary microbiome dynamics, and progression risk.** This schematic depicts a hypothesis-generating three-phase framework. During the induction phase, intravesical BCG may coincide with changes in urinary immune activity and shifts in selected urinary taxa or balances. An adaptive resistance phase may follow, characterized by higher PD-L1 expression and compositional patterns that could reflect differences in host inflammatory tone. During the reconstitution phase, the re-emergence of specific community features (e.g., *Corynebacterium*- and Enterobacteriaceae-associated indices) together with a balanced F/B ratio was associated with favorable PFS in this cohort, whereas other post-BCG patterns were associated with earlier progression. This working model does not imply causality and requires validation in independent cohorts and mechanistic studies. BCG: *Bacillus* Calmette–Guérin; F/B: Firmicutes/Bacteroidetes; PFS: progression-free survival.

Based on these observations, we proposed a working mechanistic model linking acute BCG-induced inflammation, adaptive immune resistance, and microbiome reconstitution (Figure 7). This working model is anchored primarily on phylum-level log-ratio balances and exploratory associations; genus-level labels (e.g., *Corynebacterium*) reflect Greengenes-based assignments and may vary under updated reference databases. During the induction phase, intravesical BCG may contribute to a transient expansion of Enterobacteriaceae and activation of CD204-positive myeloid cells, producing a pro-inflammatory bladder environment that facilitates tumor killing while exerting strong selective pressure on immune and microbial communities. As this acute response progresses, an adaptive resistance phase emerges, characterized by PD-L1-high states, in which communities with lower Actinobacteria and predominance of F/B may be enriched, buffering inflammation and potentially limiting further antitumor activity. In the recovery or reconstitution phase, patients who showed a more balanced post-BCG F/B ratio together with persistence of specific Greengenes-assigned signals (including *Corynebacterium*- and Enterobacteriaceae-labeled features) tended to have more favorable PFS, whereas those with a persistently high F/B ratio tended to progress earlier.

Although hypothesis-generating and derived from a single, small cohort, this model provided a biologically coherent framework that links immune and microbiome dynamics to clinical outcomes and suggested potential points of intervention.

Clinically, these findings suggested several potential applications. First, serial urine profiling of immune transcripts and microbiome composition was technically feasible, minimally invasive, and may complement cystoscopy and cytology for monitoring patients who are BCG-treated. Second, identifying post-BCG microbiome signatures associated with progression risk may indicate that microbiome-directed interventions, such as dietary modification, probiotics, or targeted intravesical microbial manipulation, could be explored as adjuncts to BCG, paralleling efforts to modulate the gut microbiome to improve responses to systemic immunotherapy [34–37]. Third, the alignment of our data with reports of PD-L1-mediated adaptive resistance after BCG, alongside preclinical evidence that combining BCG with PD-1/PD-L1 blockade enhances antitumor activity, suggests that host–microbiome interactions should inform the design of combination immunotherapy trials for NMIBC [30, 31, 38–41].

This study had several limitations that warrant acknowledgement. First, this was a single-center cohort with a modest sample size and few clinical events, which limits statistical power and external validity and precludes robust multivariable survival modeling. Larger multicenter cohorts will be required to confirm generalizability and to support clinically deployable risk stratification. Second, this clinical observational study cannot establish causality between specific urinary taxa and BCG efficacy. Mechanistic validation—such as in vitro co-culture experiments testing whether candidate genera modulate BCG-induced immune activation—was beyond the scope of the present work and will be pursued in future studies. Third, although the median follow-up was approximately 7 years, longer observation (more than 10 years) will be important to characterize long-term recurrence patterns, particularly in small subgroups such as patients with Tis; we are continuing follow-up and plan to update outcome analyses as additional events accrue. In addition, the microbiome was characterized using 16S rRNA gene sequencing of urine, providing limited taxonomic and functional resolution and being susceptible to low-biomass contamination. Future validation should incorporate extraction blanks and sequenced negative controls and apply established contaminant-filtering workflows to mitigate low-biomass bias. Because negative-control FASTQ data were not available, we could not apply established sequencing-based contaminant-filtering workflows [19, 21, 32]. Total 16S read counts were reported descriptively; however, read counts do not quantify absolute bacterial load and can be influenced by library size, amplification efficiency, and other technical factors. Our analyses involved multiple comparisons, and we used an exploratory false discovery rate threshold of 10% for mixed-effects models. Survival analyses were exclusively univariate and should consequently be interpreted as hypothesis-generating. The absence of paired tissue or stool samples prevented us from distinguishing bladder-local from systemic microbiome effects or directly linking urinary signatures to intramucosal immune cell states. Finally, generalizability to other populations, BCG strains, and maintenance regimens remains uncertain.

In sensitivity analyses, bootstrap intervals were extremely wide, and one feature showed sign flipping under LOO analyses, underscoring that the survival associations are statistically unstable in this small cohort and should be treated as exploratory signals requiring independent validation.

In addition, taxonomic classification relied on the Greengenes 13_8 reference database; therefore, genus-level assignments may be imperfect and could change with updated databases (e.g., SILVA). We accordingly emphasize phylum- and family-level patterns and balance-based features, and we interpret genus-level findings as exploratory.

Future studies should validate these urinary immune-microbiome signatures in larger multicenter cohorts with harmonized sampling and analytic pipelines. In parallel, targeted mechanistic experiments (e.g., co-culture systems incorporating BCG and candidate urinary genera) will be needed to test causality and to identify actionable pathways. Continued longitudinal follow-up will also enable evaluation of long-term recurrence and progression patterns.

In summary, we found that intravesical BCG was associated with coordinated increases in urinary immune transcripts and with modest, largely compositional stability of the urinary microbiome in this exploratory cohort. Integrated urine immune-microbiome profiling may help generate testable hypotheses and may inform future validation studies aimed at improving risk stratification and the durability of BCG therapy in NMIBC.

## Abbreviations

ASVs: amplicon sequence variants
BCG: *Bacillus* Calmette–Guérin
F/B: Firmicutes/Bacteroidetes
HG: high grade
IQR: interquartile range
LG: low grade
LOO: leave-one-out
NMIBC: non-muscle invasive bladder cancer
OS: overall survival
PD-L1: programmed death-ligand 1
PFS: progression-free survival
RFS: recurrence-free survival
TURBT: transurethral resection of bladder tumor
UTI: urinary tract infection

## Supplementary materials

The supplementary figures for this article are available at: https://www.explorationpub.com/uploads/Article/file/1002365_sup_1.pdf. The supplementary tables for this article are available at: https://www.explorationpub.com/uploads/Article/file/1002365_sup_2.xlsx.

## Declarations

### Availability of data and materials

Raw 16S rRNA gene sequencing reads have been deposited in the NCBI Sequence Read Archive (SRA) under BioProject PRJNA1413953. Other datasets generated for this study are available from the corresponding author on reasonable request.

### Copyright

© The Author(s) 2026.
