# Supplementary material for: Changes of urinary immunity and microbiome after intravesical BCG therapy and their association with outcomes in NMIBC
Source: Explor Target Antitumor Ther. 2026 Apr 13;7:1002365. doi: 10.37349/etat.2026.1002365 (PMC13087446; doi:10.37349/etat.2026.1002365)
Supplement: Supplementary file 1 [file 1002365_sup_1.pdf]

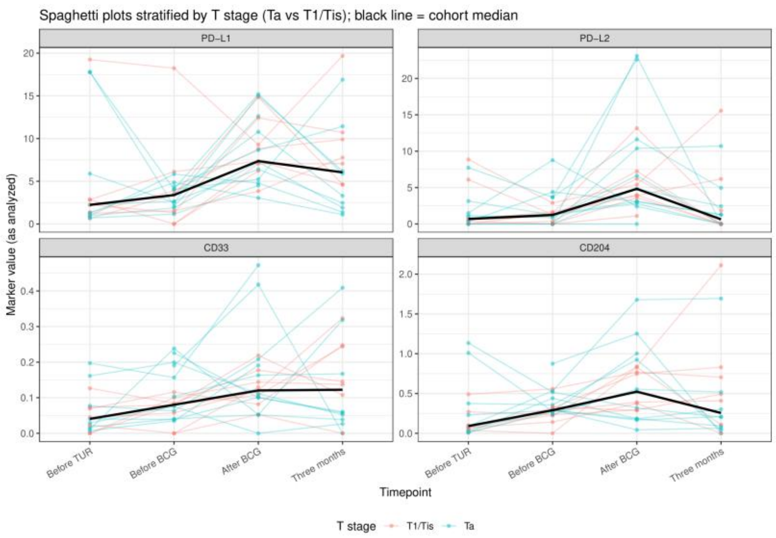

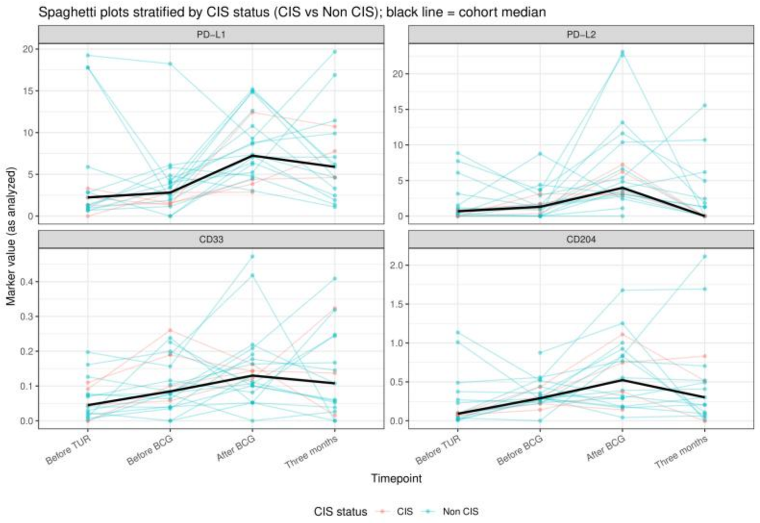

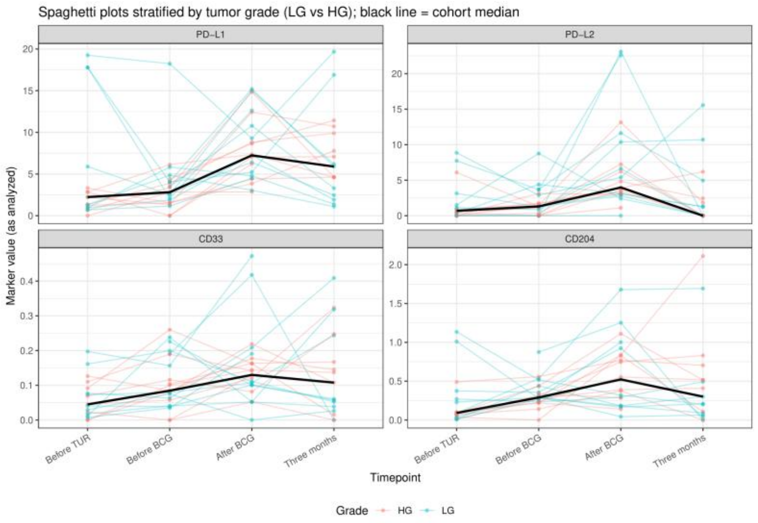

Supplementary Figure S1. Spaghetti plots stratified by CIS (CIS vs Non CIS).

Patient-level spaghetti plots of urinary immune transcript markers across four time

points, stratified by (A) pathologic stage (Ta vs T1/Tis), (B) tumor grade (LG vs HG), and (C) CIS status (CIS vs non-CIS). Each thin line represents an individual patient; the thick black line indicates the cohort median at each time point. These stratified visualizations are descriptive sensitivity analyses intended to assess whether overall longitudinal patterns are consistent across clinicopathologic strata; subgroup sizes are small and no definitive subgroup inference is intended.

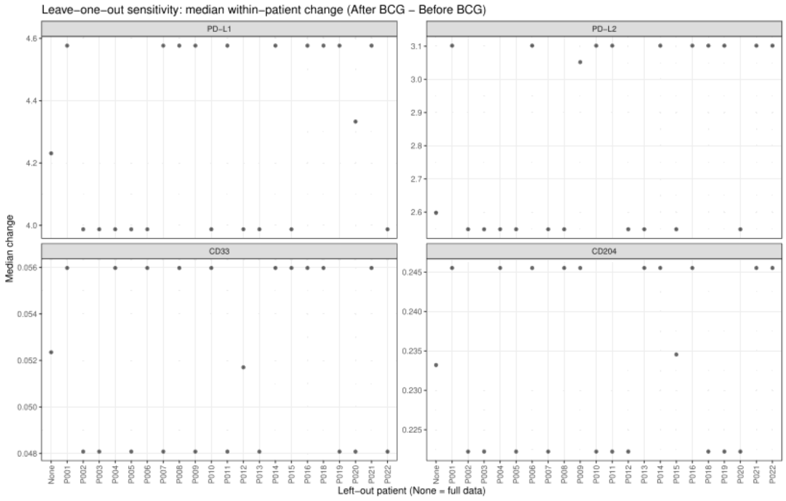

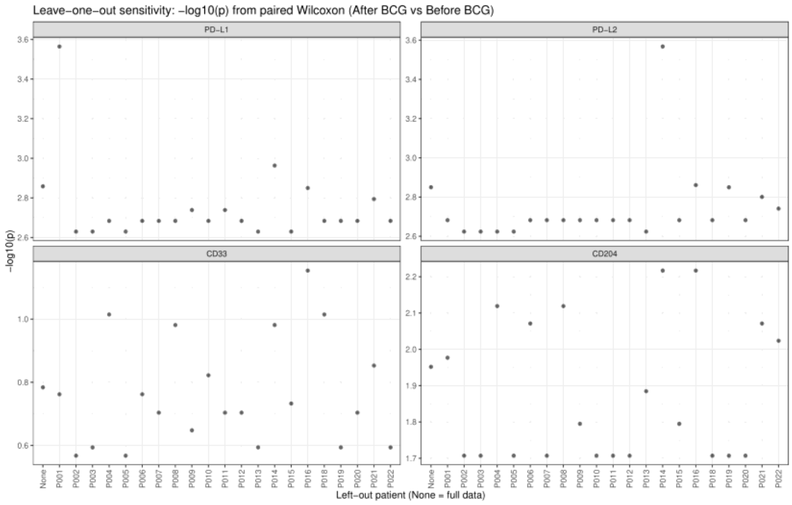

Supplementary Figure S2. Leave-one-out sensitivity analysis for the paired comparison of After BCG vs Before BCG. For each marker, we recalculated (top) the median within-patient change (After BCG − Before BCG) and (bottom) the paired Wilcoxon signed-

rank test P value after removing one patient at a time. “None” indicates the full dataset. This analysis evaluates whether the observed direction and statistical significance are dependent on any single participant.

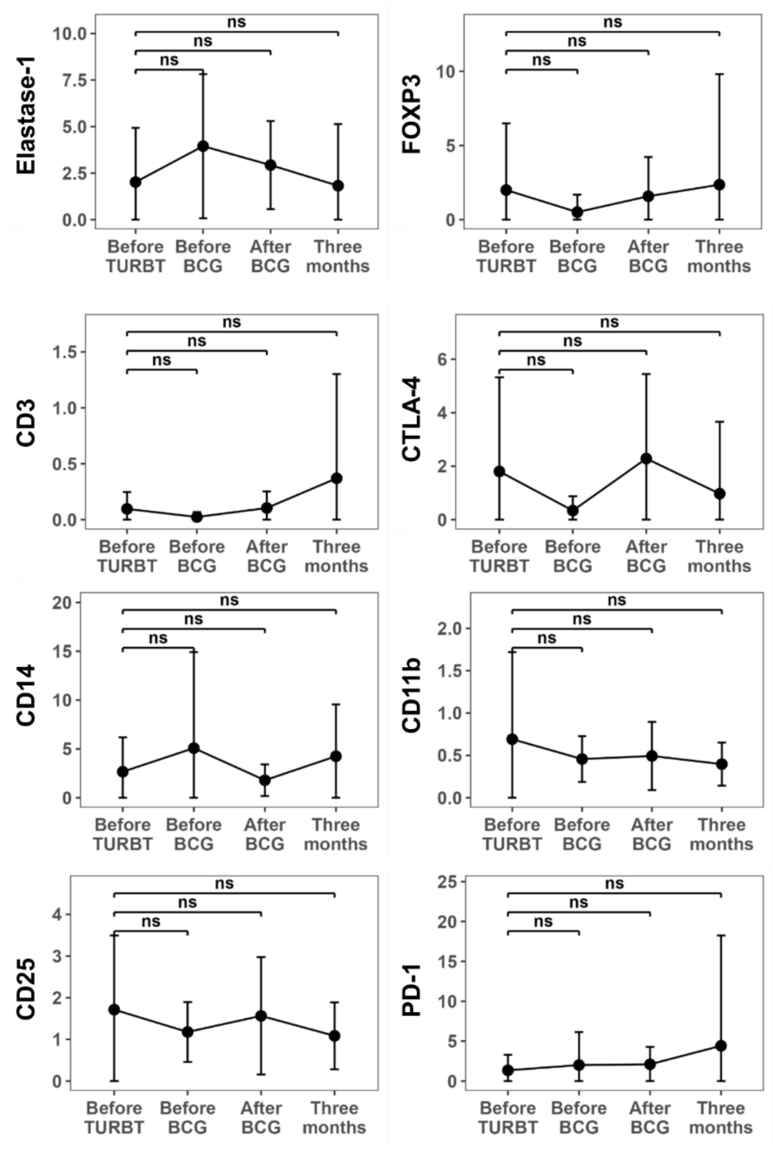

Supplemental Figure S3. Time-course changes of urinary immune cell marker mRNA expression. ΔCt values (relative to GAPDH) for CD14, CD11b (ITGAM), CD3E, FOXP3, Treg-associated marker (CD25/ISG20), Elastase-1, PD-1, and CTLA4 at four time points (Before TURBT, Before BCG, After BCG, and 3 months after BCG).

No statistically significant changes over time were detected for these markers in paired comparisons with the Before TURBT baseline.

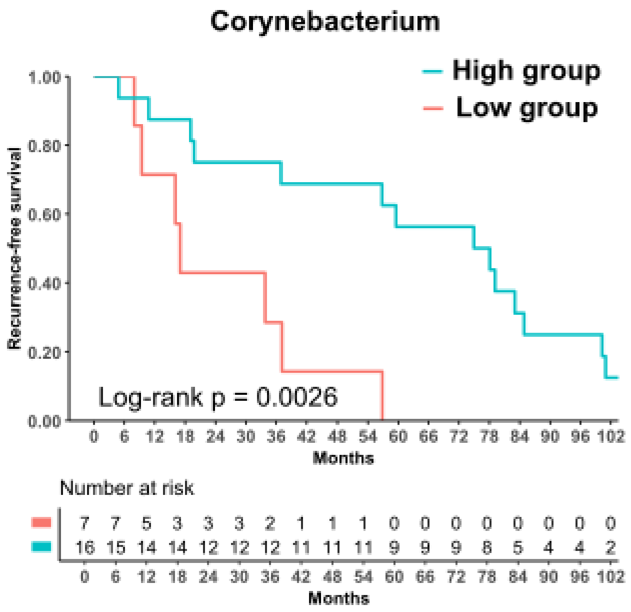

Supplementary Figure S4. Prognosis analysis: TUR and Before BCG treatments. Recurrence-free survival (RFS) curves stratified by quantile-based high versus low groups of the preoperative Corynebacterium log abundance index (Pre_Cory_nb). Patients with higher preoperative Corynebacterium abundance experienced more favorable RFS (log-rank P = 0.0026).

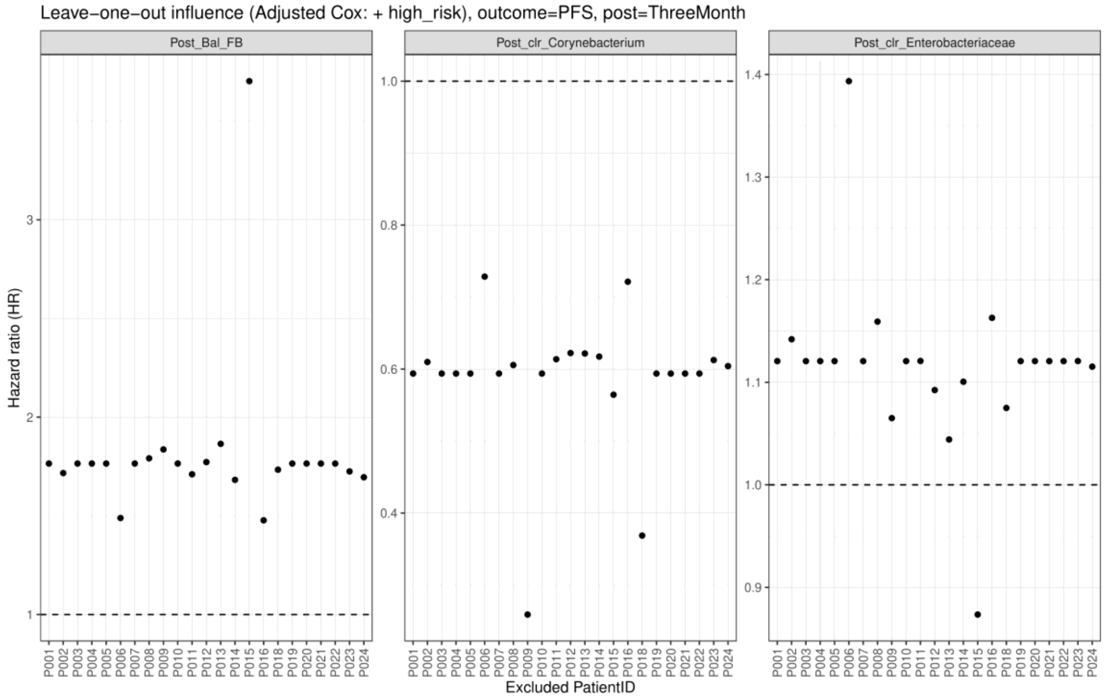

Supplemental Figure S5. Leave-one-out influence analysis for adjusted Cox models of PFS. Each panel shows the hazard ratio (HR) of the indicated three-month post-BCG microbiome feature from the adjusted Cox model including high_risk (Ta vs non-Ta), refitted after excluding one participant at a time (x-axis: excluded participant ID). Points represent the resulting HR estimates for each leave-one-out fit. The dashed horizontal line indicates HR = 1. This analysis evaluates whether the estimated association is disproportionately driven by any single participant.
